# Characterizing long-term outcomes following AMA discharges after assault-related penetrating trauma

**DOI:** 10.5249/jivr.v16i1.1875

**Published:** 2024-01

**Authors:** Ted J. Chung, Rachel M Nygaard, Ellie Moon, Logan Peter, Peter Bodurtha, Tyler Winkelman, Derek C Lumbard

**Affiliations:** ^*a*^ Department of Surgery, Hennepin Healthcare, Minneapolis, MN USA.; ^*b*^ Department of Surgery, Hennepin Healthcare Research Institute, Minneapolis, MN USA.; ^*c*^ Health, Homelessness, and Criminal Justice Lab, Hennepin Healthcare Research Institute, Minneapolis, MN USA.; ^*d*^ Department of Medicine, Hennepin Healthcare, Minneapolis, MN USA.

**Keywords:** Penetrating trauma, Violence, Reinjury, Discharge against medical advice

## Abstract

**Background:**

Patients discharged against medical advice do not receive adequate treatment and have a greater risk of readmission. This study assessed the rate of discharges against medical advice following assault-related penetrating trauma, with secondary aims to evaluate long term pre/post-injury hospitalizations and mortality.

**Methods:**

Adult assault-related penetrating injuries admitted to a Level 1 Trauma Center were identified in the prospectively maintained database. Chart review was conducted for hospitalizations ± 5 years from index injury and statewide mortality data was used to identify deaths outside of hospital care.

**Results:**

Out of a total of 1,744 assault-related penetrated injuries, 3.2% (52/1630) of survivors dis-charged against medical advice. Reasons for discharge against medical advice included: unknown (38%), home/child/family/pets (25%), unhappy with care/restrictions (23%), and work/money/other (13%). Post-discharge mortality did not differ between routine (6.5%) and against medical advice discharge (3.9%). Against medical advice and routine discharge had similar rates of any hospitalization (38.5 v 28.2%) and trauma hospitalization in prior 5-years (35 v 36%). However, significantly more against medical advice discharges had prior hospitalizations involving drug or alcohol abuse (65 v 38%), but not mental health diagnosis (55 v 55%). Significantly more against medical advice discharges have post-injury hospitalizations compared to routine discharges (48 vs 26.5%); however, include similar rates of repeat traumatic injury (36 v 32%).

**Conclusions:**

Those with against medical advice discharges were significantly more likely to have prior hospitalizations involving drug or alcohol abuse and significantly higher rates of post-injury hospitalizations. However, we did not see an increase in repeat traumatic injury or post-discharge mortality in those with against medical advice discharges when compared to those with routine discharges.

## Introduction

The incidence of penetrating trauma in the United States rose from 8% to 11% compared to pre-pandemic levels.^1^ Although the precise reasons for this increase remain elusive, hypotheses include factors such as psychosocial stress and increased substance use attributable to the pandemic.^2^ A surge in violent injuries has led to a higher frequency of unscheduled emergency department visits, often re-sulting in hospital admissions.^3^ Caring for injured persons has several barriers including a higher risk of discharging against medical advice (AMA).

Current estimates indicate that about 1.8% of trauma patients are discharged AMA, with some studies reporting rates as low as 0.76%.^4,5^ Patients who leave the hospital prematurely are at a heightened risk of incomplete treatment and subsequent readmission.^4^ Studies have identified an almost twofold increase in 30-day all-cause readmission rates for patients discharged AMA compared to those who undergo traditional discharge processes.^6^ This pattern of discharge not only leads to disjointed care but also incurs substantial healthcare expenses annually.^6^

Nearly half of the patients who suffer violent injuries experience reinjury.^7,8^ Those initially presenting with penetrating trauma are disproportionately more likely to sustain similar injuries upon subsequent visits.^7,8^ Traumatic reinjury is costly, with single-center estimates reaching $2.5 million and $1.2 million for urban and rural trauma centers, respectively.^9^ Beyond the financial impact, reinjury adversely affects patient mortality and morbidity with each additional hospital admission.^8,9^

While some research has been devoted to examining traumatic reinjury and AMA discharges within the trauma cohort, studies focused on these issues in the assault-related penetrating injury population are limited. This study aims to ascertain the rates of AMA discharges following penetrating trauma admissions and to explore whether there are discernible differences in outcomes compared to those who are discharged through standard protocols. Although the rates of AMA discharges post-penetrating trauma may align with those observed in the broader trauma population, we hypothesize that individuals that discharge AMA likely face increased rates of readmission, post-injury complications, and recurrent traumatic injuries compared to routine discharged penetrating trauma.

## Methods 

This study is a retrospective analysis of assault-related penetrating injury hospitalizations at a Level 1 Trauma center in a major city. After obtaining IRB approval, the prospectively maintained trauma databases was queried from Jan 1, 2014 to Dec 31, 2021 for assault-related penetrating injury admission encounters by using ICD 9/10 codes. We limited the dates for chart review to have a minimum of 1-year follow-up for each assault-related penetrating traumatic injury. Registry databases include patients that meet criteria for inclusion defined by the American College of Surgeons National Trauma Data Standards.^10^ Only patients captured by the trauma registry were included in the cohort. Patients who were treated in the Emergency Department (ED) and expired or expired on arrival were included in analysis. Patients treated in the ED and released or eloped from the ED during evaluation were not included in analysis due to limitations in the hospital registries. Patients with multiple assault-related penetrating injuries were included, but only their first injury was included in the cohort. Any additional injury was included in the follow-up period. After patients were identified in the registry (N=1,810), chart review was conducted. Patients who did not sustain penetrating traumatic injuries, were under the age of 18, or did not have a documented discharge disposition were excluded from the cohort (N=6). 

The dataset included age, race/ethnicity, sex, insurance type, injury mechanism, injury severity score (ISS), intensive care unit (ICU) length of stay, discharge disposition. Race/ethnicity was defined as non-Hispanic white, non-Hispanic Black, Hispanic or Latino, Asian, Native American, and other/unknown. Insurance status was categorized as private, Medicaid/Medicare, other/unknown, and self-pay. Mechanism of injury was categorized as either firearm or stabbing related injury. Discharge disposition was defined as AMA, acute care hospital, morgue, home with no aid, home with aid, inpatient rehab/psych, jail/law enforcement, long-term care facility, skilled nursing facility, or other. Charts were reviewed for 5 years prior to and up to 5 years following the assault-related trauma. This focused on hospitalizations for alcohol intoxication, substance abuse, mental health/psychiatric, and assault (any type). Outside hospital mortality was identified through publicly available death records in the state of Minnesota. Categorical variables were assessed with Chi2 test and continuous variables were assessed with student’s T-test. In Table 1 this compared characteristics of admissions between routine discharge and AMA discharge. Those that died from their injuries were included in the table for complete description of the cohort included, but not included in statistical analysis as they did not have the opportunity to discharge AMA. In Table 2, this compared factors prior to and following assault-related penetrating trauma between routine discharge and AMA discharge. Effect size was calculated using Cohen’s d. Statistical analyses were performed using Stata 15.1 (StataCorp, College Station, TX).

**Table 1:** Characteristics of patients admitted with assault-related penetrating trauma.

	Routine Discharge	AMA Discharge	Died	
N (percent)	1578 (90.5)	52 (3)	114 (6.5)	P valuea (Cohen’s d)
Age, mean (SD)	32.1 (11.5)	31.8 (10.9)	33.4 (12.3)	0.842 (0.03)
Male, N (%)	1329 (84.2)	49 (94.2)	100 (87.7)	0.051
Race, N (%)				0.002
Hispanic	119 (7.5)	2 (3.9)	4 (3.5)	
Non-Hispanic Asian	21 (1.3)	0 (0)	1 (0.9)	
Non-Hispanic White	264 (16.7)	4 (7.7)	17 (14.9)	
Non-Hispanic Black	996 (63.1)	33 (63.5)	67 (58.8)	
Non-Hispanic Native American	137 (8.7)	8 (15.4)	6 (5.3)	
Other	21 (1.3)	1 (1.9)	3 (2.6)	
Unknown	20 (1.3)	4 (7.7)	16 (14.0)	0.367
Insurance Status, N (%)				
Medicaid/care	995 (63.1)	32 (61.5)	46 (40.4)	
Other/Unknown	26 (1.7)	2 (3.9)	3 (2.6)	
Private	363 (23.0)	9 (17.3)	20 (17.5)	
Self	194 (12.3)	9 (17.3)	45 (39.5)	
Injury Severity Score, N (%)				0.815
<= 8	869 (55.1)	29 (55.8)	1 (0.9)	
9 – 15	360 (22.8)	14 (26.9)	1 (0.9)	
16 – 24	176 (11.2)	5 (9.6)	7 (6.1)	
25 - 75	171 (10.9)	4 (7.7)	105 (92.1)	
LOS days, mean (SD)	4.6 (7.9)	2.7 (3.7)	2.5 (6.9)	0.082 (0.25)
ICU stay, N (%)	335 (21.2)	11 (21.2)	28 (24.6)	0.990
ICU LOS, mean (SD)	5.9 (9.3)	3.6 (4.2)	6.9 (12.9)	0.411 (0.25)
Mechanism, N (%)				0.975
Firearm	907 (57.5)	30 (57.7)	93 (81.6)	
Stab	671 (42.5)	22 (42.3)	21 (18.4)	
HVIP, N (%)	93 (5.9)	2 (3.8)	2 (1.8)	0.766
Disposition, N (%)				<0.001
Home	1419 (89.9)	0 (0)	0 (0)	
AMA	0 (0)	52 (100)	0 (0)	
Another Hospital	4 (0.2)	0 (0)	0 (0)	
Home with service	11 (0.7)	0 (0)	0 (0)	
Inpatient Rehab/Psych	83 (5.3)	0 (0)	0 (0)	
Jail	28 (1.8)	0 (0)	0 (0)	
Long Term Care Facility	17 (1.1)	0 (0)	0 (0)	
Skilled nursing facility	10 (0.6)	0 (0)	0 (0)	
Unknown	6 (0.4)	0 (0)	0 (0)	
Died	0 (0)	0 (0)	114 (100)	

a Categorical variables were assessed with Chi2 test and continuous variables were assessed with student’s T-test. Effect size was calculated with Cohen’s d. Abbreviations: AMA, against medical advice; HVIP, hospital based violence intervention program; ICU, intensive care unit, LOS, length of hospital stay.

**Table 2:** Characteristics of hospital care 5 years prior to first assault related penetrating injury.

	Routine DC	Discharge AMA	P-value (Cohen’s d)
Prior to injury	N=445	N=52	0.107
Prior admissions (yes), N (%)	445 (28.2)	20 (38.5)	
Prior admission days before trauma, mean (SD)	-507 (481.9)	-371.15 (335.9)	0.213 (0.33)
Prior trauma admissions, N (%)	160 (36.0)	7 (35.0)	1
Prior mechanism, N (%)			0.113
blunt	118 (26.5)	3 (15.0)	
gun	15 (3.4)	0 (0)	
other	292 (65.6)	14 (70.0)	
stab	20 (4.5)	3 (15.0)	
Prior admission intent, N (%)			0.977
accident	3 (1.9)	0 (0)	
assault	150 (93.8)	7 (100)	
legal	2 (1.3)	0 (0)	
self	2 (1.3)	0 (0)	
unknown	3 (1.9)	0 (0)	
Prior admission type, N (%)			0.315
Hospital	55 (12.4)	4 (20)	
ED	390 (87.6)	16 (80)	
Prior admission mental health, N (%)	244 (54.8)	11 (55.0)	0.988
Prior admission drug or alcohol, N (%)	170 (38.1)	13 (65.0)	0.016

a Categorical variables were assessed with Chi2 test and continuous variables were assessed with student’s T-test. Effect size was calculated with Cohen’s d. Abbreviations: AMA, against medical advice; ED, emergency department.

## Results

Over the 8-year study period, we identified a total of 1,744 assault-related penetrating injury admissions at one Level 1 trauma center. Of the total cohort, 1,030 were from firearm-related injuries and 714 were from stabbing-related injuries (Table 1). Mortality during hospitalization was 6.5% of the total cohort, with firearms accounting for 93 deaths and stabbings for 21. Among survivors, 3.2% (52/1,630) were discharged against medical advice (AMA). Firearms were associated with a higher mortality rate (9%) compared to stab injuries (2.9%), yet discharge AMA rates were similar for both mechanisms - 2.9% for firearms and 3.1% for stabbings. The reasons for AMA discharges varied, with the majority being unspecified (39%), followed by personal obligations (25%), dissatisfaction with care (23%), and economic/work-related issues (13%).

Demographically, the AMA and routinely discharged groups had a mean age in the early thirties and were predominantly male (Table 1). Non-Hispanic Black patients were the most represented, followed by Non-Hispanic White, Non-Hispanic Native American, Hispanic, Asian, and Other. The proportions of patients identified as Hispanic and Non-Hispanic White were higher in the routine discharge cohort, while the proportion of Non-Hispanic Black patients was similar in the two cohorts. The proportion of Non-Hispanic Native American was significantly higher in the discharge AMA group (8.7% vs 15.4%, P<0.001). Over half of both groups had minor injuries, with an Injury Severity Score (ISS) of less than 9, and approximately 21% in each group necessitated ICU care. Enrollment in our hospital-based violence intervention program (HVIP) was higher among routine discharges compared to AMA discharges (5.9 vs 3.8%, respectively).

Hospitalization rates in the 5 years prior to index injury were similar between AMA and routine discharge when looking at any hospitalization (38.5 vs 28.2%, P=.107) and trauma hospitalization (35 vs 36%, P=.931) (Table 2). Those with prior trauma admissions were more likely to be admitted for an assault-related injury regardless of routine or AMA discharge. There was no difference in the number of days for a prior admission leading up to their index trauma admission, 500 vs 371, for routine vs AMA discharge, respectively. However, significantly more discharge AMA patients had prior hospitalizations involving drug or alcohol abuse (65 v 38%, P=.016), but not mental health diagnosis (55 v 55%, P=.988). 

Significantly more discharge AMA patients had post-injury hospitalizations when compared to routine discharge (48 vs 26.5%, P=.001); however, both groups had similar rates of repeat all cause traumatic injury (32 v 36%, P=.676) Table 3). Regardless of discharge type, there was no difference in the number of days between their initial discharge and admission with a new trauma. For both discharge types, the intent behind their post trauma admission was more likely to be assault related. A higher proportion of patients who discharged AMA required hospital admission with subsequent trauma compared to those who initially discharged routine, 32% vs 18.2%, respectively. While the post-discharge mortality was higher in the discharge AMA group, this was not statistically significant (6.5% v 3.9%, P=.447). 

**Table 3:** Characteristics of hospitalizations from 1 to 5 years following first assault related penetrating injury hospitalization.

Post injury follow-up	N=1578	N=52	P-value (Cohen’s d)
Post admissions (yes), N (%)	418 (26.5)	25 (48.1)	0.001
Post admission days from trauma, mean (SD)	397.9 (434.7)	449.8 (535.2)	0.567 (0.11)
Post trauma admission (yes), N (%)	151 (36.1)	8 (32.0)	0.676
Post mechanism, N (%)			0.99
blunt	72 (17.2)	4 (16.0)	
gun	44 (10.5)	2 (8.0)	
other	274 (65.6)	17 (68.0)	
stab	28 (6.7)	2 (8.0)	
Post admission intent, N (%)			0.744
accident	4 (2.7)	1 (12.5)	
assault	140 (93.3)	7 (87.5)	
legal	1 (0.7)	0 (0)	
self	2 (1.3)	0 (0)	
unknown	3 (2.0)	0 (0)	
Prior admission type, N (%)			0.087
Hospital	76 (18.2)	8 (32.0)	
ED	342 (81.8)	17 (68.0)	
Post admission mental health, N (%)	230 (95.0)	11 (44.0)	0.282
Post admission drug or alcohol, N (%)	180 (43.1)	14 (56.0)	0.205

a Categorical variables were assessed with Chi2 test and continuous variables were assessed with student’s T-test. Effect size was calculated with Cohen’s d. Abbreviations: AMA, against medical advice; ED, emergency department.

## Discussion

This study provides a detailed examination of long-term outcomes after penetrating injury when the patient leaves AMA. While patients with routine and AMA discharges had similar demographics and injury characteristics, those with discharges AMA had increased readmission rates, more likely to have repeat penetrating trauma, and prior hospitalizations for substance/alcohol abuse. Our findings suggest that this population is extremely vulnerable and further studies are needed to focus on addressing the barriers the prevent patients from getting completed cares. One way our institution is focusing on this population is through our hospital-based violence intervention program (HVIP). HVIPs are embedded within hospitals and provide wrap around services for survivors of violent injury. This includes support during the hospital stay and extends after discharge when clients return to the community. The services they provide may include addressing unstable housing, neighborhood safety, food insecurity, transportation, and support for on-going medical cares. Previous research has shown that HVIP implementation is an effective measure to impact traumatic reinjury.^11,12^ Informing HVIP services of this vulnerable population could help address the barriers that lead to patients discharging AMA leading to improved clinical outcomes, decreased post admission hospitalization, and decreased healthcare costs. 

In this study, 3.2% of patients hospitalized with penetrating injuries discharged AMA. Previous studies including both single center and databases have shown a range of AMA discharge rates from 0.76-8%, however this was in the general trauma population.^4,13-15^ Understanding circumstances and events leading up to individuals' injury has the potential to find modifiable factors. While we did not see a difference between prior any hospitalizations and trauma hospitalizations when looking at routine vs AMA discharge, we did find that substance and/or alcohol abuse admissions were more common in patients who would eventually discharge AMA after penetrating injury. Multiple studies in the general trauma population show that active substance and/or alcohol use at time of admission was a common reason and strong predictor for leaving AMA.^13,14^ This could serve as an opportunity for intervention through our licensed addiction medicine counselors to connect patients with outpatient resources, but the likely limited length of those hospitalizations likely creates a barrier to meaningful impact. Moreover, we found that approximately 10% of patients from each routine and AMA discharge groups had prior trauma admissions. This may present an opportunity to prevent a reinjury event. 

Similar to previous studies, we found that a majority of patients with penetrating injuries were young, non-Hispanic Black males.^16-18^ In our cohort, we had a small, but notable higher portion of Non-Hispanic Native American patients discharging AMA. There is little literature reporting outcomes following traumatic injury in the Non-Hispanic Native American population, however outcomes tend to be worse compared to the Non-Hispanic White population.^19-21^ This, in part, may be due to inconsistencies in documentation of Tribal affiliation in trauma registries. One study showed a 70% under-estimation of Native Americans in their state-wide trauma registry when connecting that data to Tribal Registry.^21^

Insurance status has long been shown to impact outcomes following traumatic injury.^18,22-25^ Only 12.4% of patients in this cohort were identified as self-pay insurance status compared to 26.8% and 48.5% reported in other firearm injury studies.^18,26^ This finding may be more related to our state-specific legislation that provides Emergency Medical Assistance and more recently Medicaid expansion under the Affordable Care Act.^27^ Ross et al. found that Medicaid expansion decreased the uninsured rate in the firearm injury population resulting in increased access to post-hospital rehabilitation and an overall reduction in in-hospital mortality.^28^ Interestingly, two studies using national trauma data showed that the strongest predictor for leaving AMA was lack of insurance.^13,15^

Multiple studies have described readmissions after firearm or penetrating trauma stemming from complications of their index injury and even with new injuries.^26,30^ Interestingly, we may be underestimating our post-injury hospitalizations with Parreco et al. finding that 30% of patients present to a different hospital after being discharged from a penetrating injury.^29^ Our findings of significantly higher post-injury hospitalizations in the populations that discharged AMA are supported by findings from Rattan et al., although they looked at all-cause firearm injuries. We also found that those who discharged AMA were at higher risk of repeat penetrating injuries. Increased risk of repeat violent injury after a hospitalization or evaluation in the Emergency Department has been established in numerous studies.^16,26,29,30^ This suggests that when leaving the hospital AMA, patients may be returning back to an environment where they engage in high risk activities and are predisposed to further trauma. Bonne et al. found that over half of patients with repeat firearm injuries were either treated and released from the emergency department or had a short hospital stay of less than 48 hours.^16^

While the data in limited and a small cohort, we chose to include our HVIP numbers in this study for a few reasons. Our HVIP was formed in 2016 with limited data collection for the first few years that was separate from our trauma registry data. This study period started in 2014, thus many of the penetrating injury patients would not have been contacted with our HVIP for services. This explains our low enrollment numbers for those that discharge routine (5.9%) and AMA (3.8%). While there are multiple hospitals that have well established HVIPs who have led the way, a majority of institutions with HVIPs remain in their infancy but it is becoming standard at most trauma centers in the U.S. These findings will be used to directly inform our HVIP to identify opportunities to increase reach and effectiveness within this specific population. This could further be used as a framework by other trauma centers with HVIPs to better understand some of the longer-term outcomes of leaving the hospital before care is completed. Importantly, being able to leverage our HVIPs to better understand self-identified needs for patients admitted to the hospital with the use of credible messengers could address many of the reasons that patients leave before their care is complete or at least help us better understand, in 39% of cases, why someone left the hospital AMA. It seems reasonable that addressing some of the basic needs we describe would allow our patients to better focus on their own healing while in the hospital.

This study has several limitations. First, this is a single center study in a metropolitan city with multiple level 1 and 2 trauma centers. Due to the small sample size, there is risk for Type 1 error. Patients presenting to other hospitals after discharging AMA or with new traumatic reinjury would not be captured in this study. Second, while we are using data from a single center, we did not include data outside our inpatient trauma registry so any patient seen by the emergency department and discharged would not be included for primary analysis. While we included an evaluation of ED visits in our pre and post 5-year chart review, we may still be underestimating discharges AMA as well as post-injury hospitalizations. Third, we may have missed post-injury mortality events outside of the hospital if the patient left the state and was thus not captured by our single state mortality data files. Finally, there are likely encounters that were not captured within our study time frame limiting our scope to study this population.

Our study provides a description of those discharging AMA following admission for penetrating trauma. These individuals remain at high risk for repeat injury and post-injury hospitalizations. Better understanding of these discharges AMA can provide HVIPs opportunities to intervene with these individuals to either prevent them from leaving before their medical treatment is complete or provide increased outpatient support after leaving. 
